# Troubleshooting of the determination of bisphenol A at ultra-trace levels by liquid chromatography and tandem mass spectrometry

**DOI:** 10.1007/s00216-015-9215-z

**Published:** 2015-12-03

**Authors:** Kamila Wilczewska, Jacek Namieśnik, Andrzej Wasik

**Affiliations:** Department of Analytical Chemistry, Chemical Faculty, Gdansk University of Technology, G. Narutowicza 11/12 Str, 80-233 Gdańsk, Poland

**Keywords:** Bisphenol A, Background contamination, Troubleshooting, LC-MS/MS system blank

## Abstract

Determination of trace amounts of bisphenol A (BPA) may cause problems mainly related to the presence of BPA in solvents (even in LC-MS grade), laboratory vessels, and plastic equipment used for sample preparation. Variable and sometimes significant amounts of BPA present in the background cause problems in obtaining good repeatability of measurements at the ultra-trace levels. Such observations (i.e., poor repeatability of results) were made during development of the LC-MS/MS method for determination of BPA in human serum samples. The method included gradient separation of the sample’s constituents. The BPA peak was present in the chromatograms not only when procedural blanks were injected but also when void injections were made. One of the possible ways to eliminate background contamination is to change the source of solvents, use a different water purification system, and introduce rigorous equipment cleaning procedures. However, despite the use of these recommended guidelines, the peak of BPA was still present in the system blank. It was observed that the intensity of the BPA peak was proportional to the time of column conditioning. It was concluded that BPA, present in the components of the mobile phase, is being enriched in the front of the separation column during its conditioning (i.e., when mobile phase elution strength was low). This paper describes effects of gradient and isocratic elution conditions on LC-MS/MS system blank. The problem of spurious BPA peak, originating from the mobile phase, was solved by replacing gradient with isocratic elution mode. The use of isocratic elution conditions with the mobile phase of relatively high elution strength (50 % of acetonitrile) allowed elimination of the peak of BPA coming from the mobile phase and significantly improved the precision of determination of BPA at low concentration levels.

## Introduction

Bisphenol A (BPA) is a high-production-volume industrial chemical mainly used in the production of polycarbonates and epoxy resins utilized in the manufacture of containers, bottles, toys, and medical devices [1]. Recent scientific findings indicate that BPA may migrate from those materials to media which are in direct contact with them [2]. Migration of BPA may occur due to hydrolysis of the polycarbonates or by the influence of a strongly acidic or basic environment. The widespread use of products containing BPA is a major source of human exposure to this compound. Toxicological studies on BPA indicate its negative health effects including endocrine-disrupting properties, even at low doses (0.01–0.71 μg/kg/day) [3]. Moreover, bisphenol A is suspected to promote the progress of many diseases [4, 5].

Determination of low concentration of BPA is a challenge for analytical chemists. Trace amounts of BPA can be present in solvents, laboratory vessels, and plastic equipment. Because of this, it is difficult to develop an analytical methodology characterized by high sensitivity and good reproducibility of results. Current procedures allow reduction of the amount of BPA in materials and reagents used in sample preparation steps. It is recommended that any contact of the sample with plastic materials be avoided during sampling and sample preparation to prevent contamination with BPA [6, 7]. Laboratory vessels should be prepared in an appropriate manner which consists of heating them at a high temperature and cleaning with ultra-pure water followed by methanol and acetone rinse [8]. Nevertheless there are situations where even these precautionary steps will not guarantee success.

The aim of this study was to investigate and troubleshoot problems encountered during determination of trace amounts of BPA in human serum samples. It was found that the source of the problem is the presence of BPA in the components of the mobile phase. Since no solvent or labware cleaning procedures were helpful in eliminating this obstacle, an investigation on other solutions has been undertaken. Our efforts resulted in a very simple way to overcome the problem of contamination of mobile phase components with ultra-trace amounts of BPA.

To the best of our knowledge, this is the first report on effects of gradient and isocratic elution conditions on LC-MS/MS system blank.

## Materials and methods

### Chemicals

BPA (≥99 %) and BPA-*d*_16_ (98 % D) were purchased from Sigma-Aldrich (Deisenhofen, Germany), and LC-MS-grade acetonitrile (ACN) was purchased from Merck KGaA (Darmstadt, Germany). Ultra-pure water was obtained with the help of an HLP 5 system from Hydrolab (Wiślina, Poland).

### Preparation of standard solutions

Stock solutions of BPA and BPA-*d*_16_ (1 mg/mL) were prepared by dissolving an appropriate amount of each substance separately in acetonitrile. The standard solutions of BPA were prepared by dilution of the stock solution in a mixture of water and acetonitrile (90:10) to obtain concentrations of 5 and 100 ng/mL. To each standard solution, an appropriate amount of BPA-*d*_16_ was added as an internal standard, to obtain a concentration of 100 ng/mL. All solutions were stored at −20 °C in glass vessels and brought to room temperature before analysis.

### LC-MS/MS analysis

The chromatographic separation was performed using an HPLC system (Nexera X2, Shimadzu, Japan) consisting of a degasser, a binary pump, an autosampler, and a thermostated column compartment. The analytes were separated on a Lichrospher C18 column (250 mm × 4 mm; 5 μm). The mobile phase was a mixture of water (component A) and acetonitrile (component B). Elution was accomplished under isocratic or gradient conditions. In the case of isocratic mode, the elution was performed using a mixture of acetonitrile and water (50:50 *v*/*v*) while the program of gradient elution was as follows: 10–90 % B (0–10 min) and 90 % B (10–12 min). The column was conditioned for 10, 20, or 30 min after each analysis. Under all conditions, the mobile phase flow rate was maintained at 1 mL/min and the temperature of the column compartment was set at 45 °C. Injection volume was equal to 10 μL. Simulations of chromatographic analyses (void injections) were performed under gradient and isocratic conditions.

Analyses were performed in multiple-reaction monitoring (MRM) mode using an LCMS-8050 triple quadrupole mass spectrometer (Shimadzu, Japan) equipped with an ESI source working in negative ionization mode. Data were collected and processed with the help of LabSolutions 5.60 SP1 software. The specific MRM transitions were chosen in flow injection mode, using standard solutions of BPA and BPA-*d*_16_. Optimum detection conditions are presented in Table 1.

**Table 1:** Optimal parameters for the monitored ion transitions and MS/MS operational parameters

Name	Pseudomolecular ion → fragment ion		Q1 prebias [V]	Collision energy [V]	Q3 prebias [V]
BPA	227.0 → 211.9		17	18	20
BPA-*d*_16_	241.0 → 142.2		26	27	13
MS/MS operation parameters					
Nebulizing gas flow [L/min]	Heating gas flow [L/min]	Interface temperature [°C]	DL temperature [°C]	Heat block temperature [°C]	Drying gas flow [L/min]
3	10	300	250	500	10

*DL* desolvation line

## Results and discussion

### LC-MS/MS system blank

**Fig. 1:**
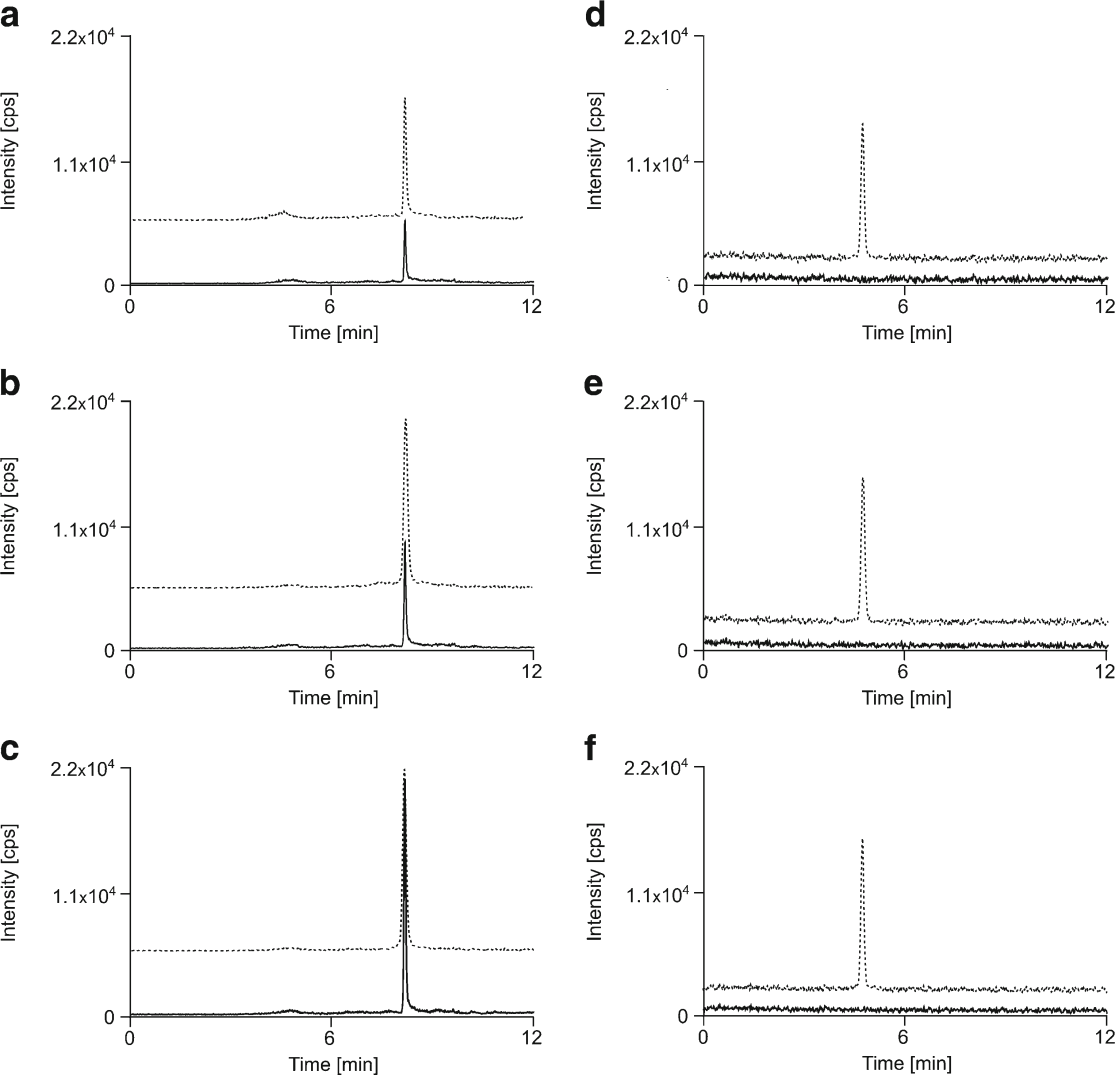
The multiple-reaction monitoring chromatograms obtained after the simulated chromatographic run (void injection) with a different time of column conditioning under gradient condition **A** 10 min, **B** 20 min, **C** 30 min and isocratic conditions **D** 10 min, **E** 20 min, **F** 30 min. *Solid line* actual chromatogram, *dashed line* BPA retention time marker (not to scale on **A**, **B**, and **C**)

Low repeatability of results especially at a low concentration level (5 ng/mL) was observed during analysis of BPA standard solutions. This was probably due to the fact that the mobile phase was contaminated with BPA. In order to verify this assumption, a series of experiments based on simulation of chromatographic analysis under gradient conditions were performed. The analytical column was conditioned for 10, 20, and 30 min. In each of these experiments, a BPA peak was observed and its area was proportional to the time of column conditioning (Fig. 1A–C). Based on these results, it was concluded that the trace amounts of BPA, present in the mobile phase, were retained on the column and later on eluted as the chromatographic gradient progressed. Retention of BPA from the mobile phase occurred during column conditioning, when the mobile phase had relatively low elution strength.

To deal with this problem, an isocratic elution mode characterized by fairly high acetonitrile content was used. It was found that the mobile phase of relatively high elution strength (50 % *v*/*v* of acetonitrile) efficiently prevents retention of BPA at the front of the chromatographic column (Fig. 1D–F). There is one drawback of this approach, however. The intensity of a chromatographic baseline is higher under isocratic conditions.

### Gradient vs. isocratic elution mode—impact on accuracy and precision of results

**Fig. 2:**
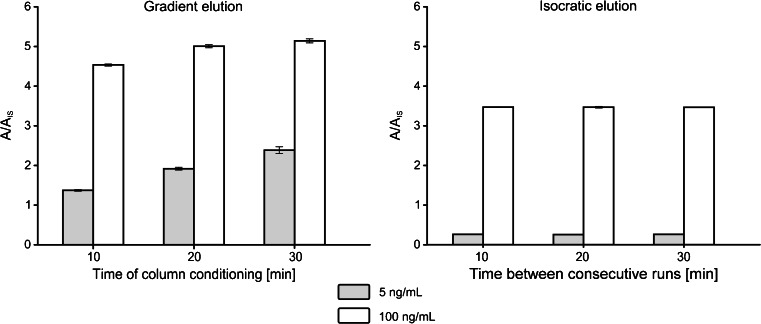
Effects of time of column conditioning (duration between consecutive runs) under gradient and isocratic conditions on *A*/*A*_IS_ values

In Fig. 2, results of triplicate analysis of two standard solutions (5 and 100 ng/mL) are shown. The chromatographic analyses were started after three different column conditioning periods (10, 20, and 30 min). The experiments were conducted under gradient and isocratic elution modes. The left-hand-side diagram (gradient elution) clearly shows the enrichment of mobile phase-born BPA. This effect is particularly well pronounced at a low concentration level (5 ng/mL). While the precision of the results obtained under the *very same* analysis conditions is acceptable, the robustness of the method will be drastically affected by even slight changes in column conditioning time, which is sometimes difficult to avoid. The experiment has shown that, at the concentration levels close to the limit of detection (1 ng/mL), the error due to change of conditioning time from 10 to 30 min may be as high as 300 %.

Under isocratic elution conditions, no such negative effects are visible. It is also worth to note that sensitivity, expressed as a Δ(*A*/*A*_IS_) vs. Δ*C* ratio, is at least two times better under isocratic elution conditions.

**Fig. 3:**
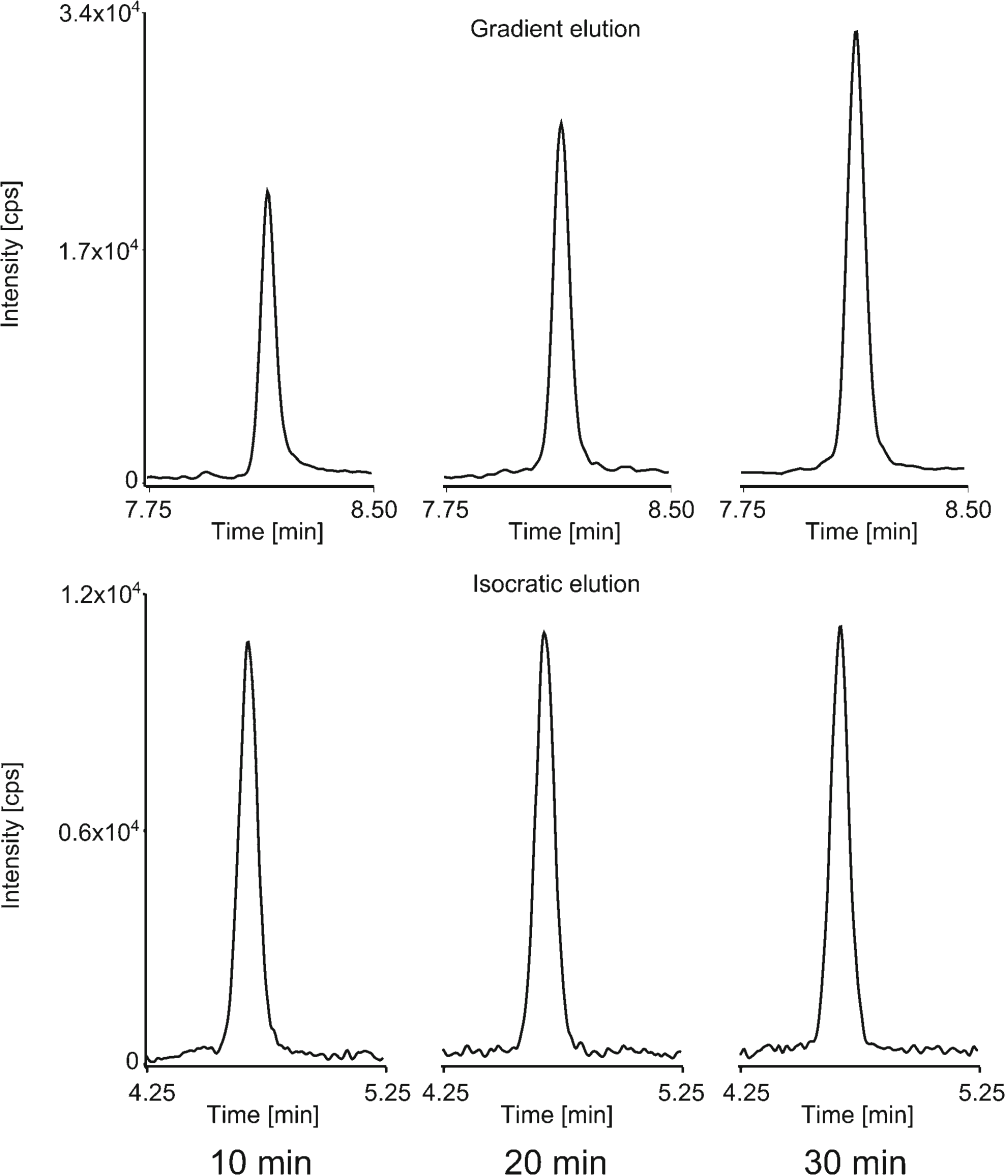
MRM chromatograms obtained after the analysis of 5 ng/mL of standard solution of BPA under gradient and isocratic conditions with a different time of column conditioning

MRM chromatograms obtained after the analysis of a standard solution (5 ng/mL) under gradient and isocratic conditions, with different column conditioning times, are shown in Fig. 3. It clearly shows superiority of isocratic elution mode over a gradient one.

## Conclusions

The presence of BPA in solvents, even in LC-MS-grade ones, may result in erratic results when trace amounts of BPA are to be determined. The problem is particularly easy to observe when a gradient elution mode is used. The BPA tends to accumulate at the front of the chromatographic column during between-run column conditioning. As a result, spurious or ghost peaks of BPA may be observed, even after blank injections. Since thorough solvent cleaning is impractical, a much better alternative is to use the isocratic elution mode. Properly chosen mobile phase composition will prevent BPA from accumulating at the front of the chromatographic column. This will result in more accurate results and a robust analytical method.
